# “Is a general belief in one’s capabilities related to mental and physical health?”: The relationships between self-efficacy, health outcomes, and health-related behaviour

**DOI:** 10.1186/s40359-026-03996-7

**Published:** 2026-03-05

**Authors:** Lucie Horelicova, Lukas Novak, Radka Zidkova, Peter Tavel, Klara Malinakova

**Affiliations:** 1 OUSHI - Olomouc University Social Health Institute, Palacký University Olomouc, Univerzitní 244/22, Olomouc, 771 11 Czech Republic; 2 Department of Community and Occupational Medicine, University Medical Center Groningen, University of Groningen, Groningen, The Netherlands

**Keywords:** Self-efficacy, Chronic conditions, Health complaints, Health-related behaviour, Risk factors

## Abstract

**Background:**

Self-efficacy, reflecting an individual’s belief in their ability to achieve goals, influences motivation, behaviour, and success. Understanding the interrelationships between general self-efficacy (GSE) and chronic conditions can contribute to developing prevention and treatment strategies.

**Methods:**

We surveyed 3,532 participants (age = 31.73 ± 13.13 years, 66% females) using an online questionnaire that assessed GSE, chronic conditions, health complaints, and health-related behaviours. Binary logistic regression analysed associations.

**Results:**

Higher GSE levels were associated with reduced odds of several chronic conditions, including pain of unclear origin (4%, 95% CI [0–8], *p* < 0.05), anxiety (6%, 95% CI [4–9], *p* < 0.001), and depression (5%, 95% CI [2–8], *p* < 0.01). Furthermore, higher GSE levels were associated with a 10% (95% CI [9, 12], *p* < 0.001) reduction in the odds of nervousness, an 8% (95% CI [6–10], *p* < 0.001) reduction in the odds of heart pounding/chest pain, and a 7% reduction in the odds of depressive feelings (95% CI [6–8], *p* < 0.001) and irritability/bad mood (95% CI [6–9], *p* < 0.001). Overall, negative associations were found with all tested health complaints except for intestinal complaints. Conversely, higher GSE levels were associated with increased odds of asthma (3%, 95% CI [0–5], *p* < 0.05) and coffee consumption (2%, 95% CI [1–4], *p* < 0.001).

**Conclusion:**

GSE was moderately associated with health complaints, chronic conditions, and health-related behaviours, suggesting its importance in the prevention and management of chronic diseases.

**Supplementary Information:**

The online version contains supplementary material available at 10.1186/s40359-026-03996-7.

## Background

Chronic conditions represent a major global threat to human health. Their pervasive and long-lasting nature necessitates urgent and sustained action from the global health community. Consequently, scientific and medical researchers remain strongly committed to investigating the aetiology of these conditions, identifying effective preventive strategies, and developing evidence-based pharmacological and therapeutic interventions [1, 2]. The onset, prevention, and treatment of chronic conditions are shaped by a wide range of determinants [3]. Many of these factors have been extensively studied, including alcohol consumption [4, 5], smoking [6, 7], physical activity [8–10], and diet [11–13]. However, several important determinants remain insufficiently explored. In recent years, growing attention has been directed toward the influence of mental health, well-being, and psychological characteristics on overall health outcomes [14–17]. One such under-examined determinant that may relate to mental health, psychological well-being, personality traits, and numerous other health-related variables is general self-efficacy (GSE) [18, 19].

Self-efficacy (SE) is an individual’s belief in their ability to achieve goals, which influences their motivation, behaviour, effort, persistence, intensity of negative emotions, level of achievement, and social environment [20, 21]. It refers to the control over events, the ability to influence one’s life, and to adapt to the situation [21, 22]. According to Bandura [21], SE is associated with certain human activities. This specific self-efficacy (SSE) is now studied in various areas of human activity, including education [23], reading [24], smoking [25], entrepreneurship [26], and social networks [27]. In contrast, Schwarzer [28] describes SE as a situation-independent and relatively enduring characteristic of a person that can serve as a dispositional resource in times of stress, incorporating all previous successes and failures. Such SE is called general self-efficacy and represents a global sense of personal capability perceived across situations and tasks [28, 29].

SE is a psychological characteristic that has the potential to influence health [30], according to the holistic bio-psycho-social model. Research has shown significant links between higher levels of SE and reduced symptoms of mental illness, as well as better management and adherence to treatment [31, 32]. Recent findings, for instance, indicate that higher SE predicts fewer daily anxiety symptoms and faster early treatment response in anxiety disorders [33]. Furthermore, higher levels of SE are consistently correlated with lower stress levels [34], which is important because chronic stress significantly contributes to the development and worsening of many mental illnesses. Conversely, low levels of SE are associated with higher levels of anxiety, depression [35, 36], stress [36, 37] and fear [38]. Some studies suggest existing relationships between SE and certain somatic chronic conditions, such as cancer [39–41], diabetes [42], hypertension [43], and asthma [44, 45].

Chronic conditions are a global problem. According to WHO [1, 46], chronic diseases kill 17 million people under the age of 70 each year, out of a total of 41 million deaths. These long-term conditions arise from environmental, socioeconomic, behavioural, biological, and psychological factors [3, 46, 47]. Environmental risk factors include, for example, air pollution, weather changes, and UV radiation [3, 48, 49]. Socioeconomic risk factors, for example, include income, age, gender, and ethnicity [3, 50]. Behavioural risk factors are, for example, smoking, excessive drinking, drug abuse [51], unhealthy eating habits [52, 53], and physical inactivity [54]. Biological risk factors include, for example, elevated arterial pressure [55] and genetic predisposition [56–58]. Psychological risk factors are most commonly represented in the literature by stress and neuroticism [59–62].

Examining the aforementioned risk factors for chronic conditions and the areas where SE can impact health, we observe interesting intersections. According to the available studies, the level of SE is associated with health-risk behaviours, such as smoking, drinking alcohol [51], quality of dietary habits [52], and amount of physical activity [54]. These behaviours are also among the main risk factors for chronic conditions [3]. From this perspective, the degree of SE is linked with some risk factors for the development of chronic conditions and could, therefore, impact their origin.

To the best of our knowledge, most published studies examine chronic conditions and health complaints in the context of SSE, not GSE. These studies typically examine the relationship between SE and chronic conditions, or the management of health complaints. A similar situation applies to the relationship between SE and health-related behaviour, with much evidence associated with SSE. We did not find studies directly addressing the link between health-related behaviour and GSE. However, identifying the associations between GSE, chronic conditions, health complaints, and health-related behaviours could influence the view on the prevention and treatment of chronic conditions and thus contribute to reducing their prevalence and mortality. Thus, our study aims to explore the relationship between GSE, selected chronic conditions, health complaints, and health-related behaviour.

## Methods

### Participants

Data were collected through an online questionnaire hosted on a platform developed by the Olomouc University Social and Health Institute (OUSHI). Our sample consisted of adult participants who volunteered to take part in the survey. Participation was conditional upon signing an informed consent form before beginning the survey. The study received ethical approval from the Faculty of Theology Ethics Committee of Palacký University in Olomouc (No. 2020/4).

Data collection was conducted between September 2022 and January 2024 using a snowball sampling method facilitated by university students. The data gathering process was incorporated into university coursework, with student participation serving the dual purpose of earning academic credits and fulfilling a core component of their educational learning objective. Students served as the initial contacts, tasked with reaching out to 10 participants from their immediate and wider social circles, motivating them to complete the questionnaire, and subsequently encouraging its further dissemination among their contacts. Of the 7,253 respondents who agreed to participate in the survey and completed basic socio-demographic data, 1,333 were excluded due to the high likelihood of having completed the questionnaire multiple times. This likelihood was calculated using the formula:

$$\:p=\frac{{\sum\limits_{i=1}^n}{x}_{ji}\ne\:0}{n}$$

In the equation, *p* represents the probability of browser type and version matching for each student, $\:{x}_{ji}$ denotes the number of rows where the i-th element from the interviewer’s code column matches the j-th element from the browser’s type and version row used to complete the questionnaire, and *n* refers to the total number of rows in the matrix. Subsequently, a tolerance limit was calculated using these match probabilities. In the equation below, *m* represents the number of questionnaires the student completes, and *q* is the student’s designation.

$$\begin{aligned}\\& more\:significant\:tolerance\:limit\\&=\underset{\_}{p}*med\left({m}_{1},{m}_{2},{m}_{3}...{m}_{k}\right)*{{\sum\limits_{q=1}^n}\left({q}_{m}>1\right)}\end{aligned}$$

Then, the responses of 2,098 people who completed the questionnaire in under 26 min were excluded, as this was the shortest amount of time deemed necessary to complete the questionnaire truthfully. Respondents were repeatedly asked about their weight, age and height. If their answers to the same question differed by more than 2 kg, years or cm, they were excluded (*n* = 380). Participants who provided identical answers to at least three distinct questionnaires forming part of the comprehensive test battery were also excluded (*n* = 207). The final sample size, therefore, consisted of 3,532 participants aged 18 to 80 (M = 31.73, SD = 13.13; females: 66%).

### Measures

Self-efficacy was measured by the Generalized Self-Efficacy Scale (GSES) [63]. The GSES consists of ten items designed to assess an individual’s perceived self-efficacy, that is, their belief in their ability to face new or challenging situations and overcome related obstacles and setbacks. For example: “It is easy for me to stick to my aims and accomplish my goals.” [63]. For each item, respondents can choose from four options ranging from not at all true [1] to exactly true [4]. The higher the total score, the greater the individual’s generalized sense of self-efficacy. The Czech version of GSES was used in this study [64]. Cronbach’s alpha was 0.88 for our sample. The GSES was used as a continuous variable in the research.

Health-related behaviour was assessed using the question: ‘How often in the past month have you smoked/drunk alcohol/used illegal drugs/drunk coffee/used the TV or computer to relax?’ Respondents selected from the following six options: never (1), about once or twice (2), about every week (3), more than once a week (4), every day (5), or many times a day (6). For analysis, responses for each item were dichotomized. The occurrence of alcohol consumption, smoking, and illegal drug use was defined by responses 2–6. The occurrence of coffee consumption and the use of TV or a computer to relax was defined by responses 5 and 6.

The prevalence of physical chronic conditions was assessed by the question, ‘Do you have a diagnosed long-term physical illness or disability?’ followed by a multiple-choice list of selected chronic conditions (allergies, arthritis, back pain, migraine, thyroid disease, ischemic heart disease, hypertension, diabetes, pelvic pain, chronic tiredness, psoriasis, obesity, skin diseases, asthma, cancer, gastric reflux, pain unclear origin, inflammatory bowel disease, chronic lung disease, stomach or duodenal ulcers, stroke, none of these conditions). The occurrence of a physical chronic condition was indicated by the respondent selecting it from the list.

The presence of psychiatric chronic conditions was measured by the question, “Do you have a diagnosed long-term psychiatric illness or disorder?” followed by a multiple-choice list of selected conditions (ADHD, paranoia, depression, paranoid schizophrenia, schizotypal disorder, anxiety), with occurrence indicated by the respondents’ selection. The included conditions were considered suitable because their symptom profiles and treatment requirements remain relatively stable over time and are not characterised by distinct episodic changes [65–69].

The frequency of health complaints (headache, stomachache, back pain, intestinal complaints, feelings of depression, irritability/bad mood, nervousness, trouble falling asleep, dizziness, sore throat/cold, heart pounding/chest pain, tingling in limbs or face) was assessed by a question ´In the past month, how often have you had the following issues?´ Respondents then chose from the following answers: never (1), about once or twice (2), about every week (3), more than once a week (4), or every day (5). For further analysis, responses for each item were dichotomised, with categories 3–5 pooled and treated as indicating the presence of the complaint.

Socio-demographic data were collected, including age, gender, education, and economic status. Age, gender, and education served as covariates in the subsequent analyses, while economic status was included for descriptive purposes only. Education was assessed using seven categorical levels, ranging from Basic school to Postgraduate – Doctorate. The specific response categories were: Basic school, Secondary vocational school, High school, Higher vocational school, Undergraduate (bachelor’s degree), Postgraduate – Master’s (M.A., M.Sc., M.Phil.), and Postgraduate – Doctorate (PhD, D.Phil., D.Sc.). Economic status was measured using the following nominal categories: student, employed, freelancer, unemployed, or retired/invalid pensioner. Gender was reported dichotomously (female or male), and age was recorded as a continuous numerical variable.

### Statistical analysis

In the first step, we tested the socio-demographic differences in the degree of GSE. The Shapiro-Wilk test revealed that our data did not meet the normality assumption; therefore, non-parametric methods were used for further analysis. The differences among the groups were tested using a Kruskal-Wallis test. If the result was statistically significant, a Wilcoxon test was performed for gender comparisons, and a Dunn’s test was performed for comparisons of education and economic status.

We used binary logistic regression to analyse the associations between GSE (independent variable) and all binomial dependent variables. During the first step, we analysed the relationship between the selected psychological and physical chronic conditions and the GSE. Further, we dichotomized the variables of health complaints (headache, stomachache, back pain, intestinal complaints, feelings of depression, irritability/bad mood, nervousness, trouble falling asleep, dizziness, sore throat/cold, heart pounding/chest pain, tingling in limbs or face) and health-related behaviours (smoking, drinking alcohol, using illegal drugs, drinking coffee, using the TV or computer to relax). Subsequently, we examined the relationships between health complaints and GSE, as well as between health-related behaviours and GSE, using binary logistic regression.

In all analyses, we adjusted for age, gender, and education. Non-adjusted results were also reported. Correction for multiple testing was performed using the Benjamini-Hochberg correction. Data were analysed using IBM SPSS Statistics for Windows, Version 21.0 (IBM Corp., Armonk, NY, USA) and the Psychtoolbox toolkit [70].

## Results

### Description of the socio-demographic characteristics of the research sample and testing sociodemographic differences in GSE

The most significant proportion of our sample was represented by employed people and individuals with a high school education. The highest GSE scores were achieved by men, freelancers, and people with a master’s degree. The socio-demographic characteristics of the research sample and the differences between selected sociodemographic factors in GSE are described in more detail in Table 1.

**Table 1:** Descriptive statistics of the study sample and Socio-Demographic comparison

Variable	*n* (%)	GSE Group difference	GSE M(SD)
Gender			
1. Man	1200 (34.0)	*p* < 0.001	29.6 (5.2)
2. Woman	2332 (66.0)		27.8 (4.9)
Economic status			
1. Student	1282 (36.3)	*p* < 0.001 (1–3***,1–4***,2–4**,3–4***,4–5***)	27.7 (5.1)
2. Retired or invalid pensioner	114 (3.2)		28.3 (5.3)
3. Employed	1744 (49.4)		28.6 (5.0)
4. Freelancer	268 (7.6)		30.5 (4.9)
5. Unemployed	124 (3.5)		27.6 (5.4)
Education			
1. Basic School	188 (5.3)	*p* < 0.001 (1–3*,1–5***,1–6***,2–6***,3–6***,4–6***,5–6*)	26.6 (5.7)
2. Secondary vocational school	338 (9.6)		28.1 (5.2)
3. High school	1830 (51.8)		28.2 (5.0)
4. Higher vocational school	137 (3.9)		27.3 (4.4)
5. Undergraduate (bachelor’s degree)	470 (13.3)		28.71 (5.0)
6. Postgraduate - master	535 (15.2)		29.9 (4.9)
7. Postgraduate - doctorate	34 (1.0)		29.5 (4.3)

*GSE* General Self-efficacy; *M* Mean; *SD *Standard Deviation

* *p* < 0.05; ** *p* < 0.01; *** *p* < 0.001. 1–3 *** and 1–4 *** indicate post-hoc pairwise comparisons between two specific groups within the analysed variable (Economic Status, Education), accompanied by an interpretation of statistical significance. The numbers correspond to the group categories (e.g., 1 vs. 3, 1 vs. 4), and this interpretation applies to all other comparisons listed

### GSE and chronic conditions

The results of the binary logistic regression for physical chronic conditions indicated that higher levels of GSE were associated with a 4% decrease in the odds of having pain of unclear origin and a 3% increase in the odds of having asthma; no significant associations were found in other cases.

The results of the binary logistic regression for psychiatric chronic conditions indicated that higher GSE levels were associated with a 6% reduction in the odds of having anxiety and a 5% reduction in the odds of having depression. No significant associations were found for other psychiatric chronic conditions. The results are described in more detail in Table 2.

**Table 2:** Binary logistic regression results of GSE and physical and psychiatric chronic conditions

Variable	Effect type		Allergies	Arthritis	Back pain	Migraine	Thyroid disease	Ischemic heart disease
GSE	crude	*OR* 95% CI	1.00 (0.99–1.02)	1.02 (0.98–1.06)	0.99 (0.97–1.01)	1.00 (0.97–1.02)	0.98 (0.95–1.01)	0.98 (0.91–1.04)
GSE	adjusted	*OR* 95% CI	0.99 (0.98–1.01)	1.01 (0.96–1.06)	0.99 (0.97–1.01)	1.01 (0.98–1.04)	0.98 (0.96–1.01)	0.96 (0.89–1.03)
Variable	Effect type		Diabetes	Pelvic pain	Chronic tiredness	Psoriasis	Obesity	Skin diseases
GSE	crude	*OR* 95% CI	1.00 (0.95–1.04)	0.98 (0.95–1.01)	0.97 (0.93–1.02)	1.02 (0.96–1.09)	1.01 (0.99–1.04)	1.01 (0.99–1.04)
GSE	adjusted	*OR* 95% CI	0.99 (0.94–1.04)	1.01 (0.98–1.04)	0.98 (0.93–1.02)	1.02 (0.96–1.09)	1.00 (0.97–1.03)	1.02 (0.99–1.04)
Variable	Effect type		Cancer	Gastric reflux	Pain of unclear origin	Inflammatory bowel disease	Chronic lung disease	Stomach or duodenal ulcers
GSE	crude	*OR* 95% CI	1.01 (0.94–1.09)	1.00 (0.97–1.04)	0.96 * (0.92–0.99)	1.03 (0.97–1.1)	1.00 (0.92–1.08)	0.95 (0.88–1.02)
GSE	adjusted	*OR* 95% CI	1.02 (0.94–1.1)	1.00 (0.96–1.03)	0.96 * (0.92-1.00)	1.03 (0.97–1.09)	1.00 (0.92–1.08)	0.95 (0.88–1.03)
Variable	Effect type		Asthma	Stroke	Hypertension			
GSE	crude	*OR* 95% CI	1.01 (0.99–1.04)	0.99 (0.86–1.15)	1.01 (0.99–1.04)			
GSE	adjusted	*OR* 95% CI	1.03 * (1.00-1.05)	0.93 (0.78–1.11)	0.99 (0.96–1.02)			
Variable	Effect type		Paranoia	Depression	Schizotypal disorder	Paranoid schizophrenia	Anxiety	ADHD
GSE	crude	*OR* 95% CI	0.92 (0.81–1.04)	0.94 *** (0.91–0.97)	0.91 (0.83–1.01)	0.96 (0.84–1.1)	0.92 *** (0.9–0.95)	0.98 (0.94–1.02)
GSE	adjusted	*OR* 95% CI	0.95 (0.84–1.07)	0.95 ** (0.92–0.98)	0.93 (0.84–1.02)	0.97 (0.85–1.11)	0.94 *** (0.91–0.96)	0.99 (0.95–1.03)

*N* = 3, 532; Online Survey; Covariates = age, gender, education

*GSE* General Self-Efficacy; *OR* Odds Ratio

95% CI – 95% Confidence Interval

* *p* < 0.05; ** *p* < 0.01; *** *p* < 0.001

### GSE and health complaints

There was a significant negative link between GSE and all health complaints examined. After adjusting for age, gender, and education, the significance of intestinal complaints was no longer evident. The strongest association was found between GSE and nervousness (OR = 0.90, *p* < 0.001). The weakest relationship was between GSE and tingling in limbs or face (OR = 0.97, *p* < 0.05). Table 3 provides more detailed results.

**Table 3:** Binary logistic regression results of GSE and health complaints

Variable	Effect type		Headache	Stomachache	Back pain	Intestinal complaints
GSE	crude	*OR* 95% CI	0.95 *** (0.93–0.96)	0.95 *** (0.93–0.96)	0.97 *** (0.95–0.98)	0.97 *** (0.95–0.99)
GSE	adjusted	*OR* 95% CI	0.96 *** (0.95–0.98)	0.96 *** (0.94–0.98)	0.98 ** (0.96–0.99)	0.98 (0.96-1.00)
Variable	Effect type		Nervousness	Trouble falling asleep	Dizziness	Sore throat/cold
GSE	crude	*OR* 95% CI	0.89 *** (0.88–0.91)	0.96 *** (0.94–0.97)	0.93 *** (0.91–0.95)	0.96 *** (0.94–0.98)
GSE	adjusted	*OR* 95% CI	0.90 *** (0.88–0.91)	0.96 *** (0.95–0.98)	0.94 *** (0.92–0.97)	0.97 ** (0.95–0.99)
Variable	Effect type		Feelings of depression	Irritability/ bad mood	Heart pounding/ chest pain	Tingling in limbs or face
GSE	crude	*OR* 95% CI	0.92 *** (0.90–0.93)	0.92 *** (0.91–0.93)	0.91 *** (0.89–0.93)	0.97 ** (0.95–0.99)
GSE	adjusted	*OR* 95% CI	0.93 *** (0.92–0.94)	0.93 *** (0.91–0.94)	0.92 *** (0.90–0.94)	0.97 * (0.95–0.99)

*N* = 3, 532; Online Survey; Covariates = age, gender, education

*GSE *General Self-Efficacy; *OR* Odds Ratio

95% CI – 95% Confidence Interval

* *p* < 0.05; ** *p* < 0.01; *** *p* < 0.001

### GSE and health-related behaviour

Binary logistic regression indicated that higher GSE levels were associated with a 3% increase in the odds of coffee consumption. No relationship was found between GSE and the use of illegal drugs, nor was one found between GSE and smoking. Furthermore, no significant associations were found between GSE and alcohol drinking, or between GSE and using a TV or computer to relax. More detailed results are described in Table 4.

**Table 4:** Binary logistic regression results of GSE and Health-Related behaviour

Variable	Effect type	Smoking	Drinking alcohol	Using illegal drugs	
GSE	crude OR (95% CI)	0.99 (0.98–1.01)	0.99 (0.98–1.01)	1.01 (0.99–1.03)	
GSE	adjusted OR (95% CI)	0.98 (0.98–1.01)	0.99 (0.97–1.01)	1.01 (0.98–1.03)	
Variable	Effect type	Drinking coffee	Using TV or a PC to relax		
GSE	crude OR (95% CI)	1.03 (1.02–1.05) ***	0.99 (0.98–1.01)		
GSE	adjusted OR (95% CI)	1.03 (1.01–1.04) ***	0.99 (0.97-1.00)		

*N* = 3, 532; Online Survey; Covariates = age, gender, education

*GSE* General Self-Efficacy; *OR* Odds Ratio

95% CI – 95% Confidence Interval

* *p* < 0.05; ** *p* < 0.01; *** *p* < 0.001

## Discussion

Our research aimed to assess the relationships between GSE and chronic conditions, health complaints, and health-related behaviours. The results showed that higher levels of GSE are associated with a reduction in the odds of having pain of unclear origin, depression, and anxiety, but also with an increase in the odds of having asthma. There was also a negative association between GSE and the health complaints studied. The only exception was intestinal complaints, for which statistical significance diminished after adjustment for age, gender, and education. Regarding health-related behaviour, only one significant relationship emerged, indicating that higher GSE was associated with increased odds of coffee consumption.

Our finding that GSE was negatively associated with pain of unclear origin is consistent with research by Petersen et al. [71], which describes existing relationships between GSE and the prevalence of functional somatic disorders. This research includes findings that higher levels of GSE reduce pain, as well as functional impairment. A similar relationship was also found by Wojeck et al. [72] in a study of patients with systemic sclerosis. According to a study by Tsuji et al. [73], higher levels of pain self-efficacy correlate with lower levels of pain-related disability, anxiety, and depression and, in the context of the fear-avoidance model, may serve as a protective factor against the vicious cycle of chronic pain. Pain self-efficacy in the aforementioned study represents a component of the fear-avoidance model that influences our response to pain [74–76]. Based on our findings, GSE could work similarly. The experience of stressful situations is one of four factors that determine adaptive or non-adaptive response strategies to pain [74]. Mastery of a stressful situation is typically associated with a reduction in stress and psychological problems, and it has also been linked to an increase in GSE [77, 78]. In turn, GSE is often suggested to be beneficial for improving adaptive coping strategies and mitigating stress [79, 80]. Thus, GSE could promote an adaptive response to pain, thereby decreasing the risk of chronic pain. For some chronic pain, the cause is not clear. Such pain may be psychosomatic in origin and, therefore, strongly influenced by stress and psychological state [81, 82]. By its positive effect on stress management, GSE may also contribute to reducing the incidence of this type of pain.

Our research has also shown a positive relationship between GSE and asthma. We found no studies examining the association between the development of asthma and GSE. However, some studies confirmed the relationship between SSE and asthma treatment and management [44, 45, 83–85]. Studies have shown that a significant trigger for asthma is primarily harmful air pollution, environmental allergens, and viruses [60–62], as well as high physical activity [86–88]. Higher levels of SE may contribute to increased physical activity and a more active lifestyle [66–70], which in turn could increase exposure to certain types of triggers [89].

Our results showed a negative relationship between GSE and anxiety and depression. Other studies also confirm that higher levels of SE are associated with a lower likelihood of depression and anxiety [36, 90–92]. Given that depression and anxiety, as psychological illnesses, are by their very nature primarily caused by psychological factors [93–97], the association of GSE with their presence is conceptually sound. The development of anxiety is closely related to stressful, traumatic, and otherwise challenging life situations, as well as interpersonal relationships and childhood shyness and nervousness, among other factors [98–102]. The social environment and interpersonal relationships also play an essential role in building SE [21, 22, 103]. Good relationships, healthy self-esteem, and individuality are linked with higher SE and resilience to harmful environmental influences and stressful situations [104, 105]. SE and resilience may promote better coping with stressful situations, thereby protecting against anxiety [36, 104, 106]. The association between higher levels of SE and a lower occurrence of anxiety and depression has been established in numerous studies [107–110]. Conversely, there are studies that suggest a link between the presence of anxiety, depression, and lower levels of SE [101, 102]. These findings indicate that it would be beneficial to focus on increasing SE in the therapies of individuals with depression and anxiety.

All health complaints examined, except intestinal complaints, were negatively associated with GSE, with the strongest relationship observed for nervousness. Other studies have already suggested a link between, for example, SE and back pain [111], headache [112], dizziness [113], mood disorders [95], and sleep disturbances [114], which is consistent with our results. Many health complaints are influenced by an unhealthy diet [115, 116], lack of exercise [117, 118], stress [119], and an overall unhealthy lifestyle [120]. The improvement of many of these health factors is associated with higher levels of SE [121–124]. Similarly, higher SE is linked to a reduction in stress and an improvement in overall well-being [109, 110, 125], which, in line with the psychosomatic approach, are considered important health factors [81, 82, 126]. Therefore, in relation to health complaints, GSE may have a positive impact on crucial behavioural and psychological determinants of health. Concurrently, it is necessary to note that a wide range of health complaints, such as stress, may be associated with lower levels of SE [127, 128]. These findings suggest that both the prevention and management of health complaints should include components focused on enhancing SE and psychological well-being.

Our results further showed a positive association between GSE and coffee consumption. We found no studies that specifically investigated this association. However, we did find research presenting the positive effects of coffee consumption on reducing stress, symptoms of depression and anxiety, and on improving health and well-being [129–132]. These relationships may contribute to explaining the association between GSE and coffee consumption. Stress, significant mental effort, and work pressure, which can lead to psychological problems, are often associated with demanding job positions frequently held by highly educated individuals [133]. These individuals not only have higher levels of GSE but also consume more coffee, according to research [134]. Caffeine in coffee can help reduce fatigue and improve concentration, in addition to other positive effects on physical and mental state [129, 135]. The positive relationship between GSE and coffee consumption can therefore be explained as a strategy for coping with high workload and fatigue.

### Strengths and limitations

The primary strength of our research is the sample size. A further contribution is that, unlike most previous studies that focus on various specific self-efficacies, we investigate the relationship between GSE and chronic conditions and health problems. We view GSE as a personality trait that may function as a psychological risk factor and play a role in the occurrence of health problems. Our research demonstrates that even lesser-known psychological factors, which are susceptible to influence, can play a protective role in maintaining health.

A significant weakness of our research is the method used for data collection. The use of a snowball sampling approach meant that a random selection of participants could not be ensured. Consequently, this design introduced a potential for selection bias and may have resulted in a sample that was not fully representative of the target population. This limitation is further exacerbated by the sample’s composition, which predominantly consists of students and employed individuals rather than those from other economic statuses. Therefore, the generalizability of our findings is constrained to the studied sample, meaning the results should be interpreted with caution when applied to a broader population.

A further limitation is the possibility of duplicate questionnaire completion. This arises from the recruitment method involving students during their practical training, increasing the likelihood that some participants were approached multiple times. To mitigate this issue, we utilized methods to detect duplicated responses in our data. We also acknowledge the reliance on self-reported data concerning health complaints. This method carries the risk of biases, notably social desirability bias or recall bias. Moreover, the respondents’ current psychological state may subtly influence their subjective assessment of both their own health and their self-efficacy. This suggests a potential for the observed associations to be somewhat exaggerated, which should be considered when interpreting the results. Finally, our research may have been influenced by the fact that multiple risk factors contribute to the occurrence of chronic conditions, which can be challenging to capture in data collection. While genetic predispositions play a crucial role in many chronic conditions, most of the conditions we studied were not primarily caused by genetics. This factor helped to reduce the risk of bias in our results. For instance, chronic lung diseases (including cystic fibrosis), which are mainly caused by a genetic mutation, showed no statistically significant association with GSE in our study.

### Implications for practice

The results of our research can raise awareness of the importance of GSE for physical and mental health. Our findings may also highlight the role of lesser-known psychological risk factors in the prevention and management of chronic conditions and their associated symptoms.

### Implications for research

Our research findings point to existing relationships between GSE, multiple chronic conditions, several health complaints associated with chronic diseases, and health-related behaviours. A closer understanding of their interrelationships would be aided by qualitative research focusing, for example, on the construction of GSE, the extent of GSE, and possible changes in people with chronic conditions. More precise insights into the development of relationships between GSE and chronic conditions could then be provided by a longitudinal study.

The associations between GSE and health complaints indicate that, alongside SSE, GSE may also potentially improve the prevention and management of chronic conditions and health problems. Further research could investigate whether, for some diseases, health complaints, behaviours and areas of human activity, the relationships found for SSE also apply to GSE. The results could help to understand the nature of the concept of self-efficacy and whether it relates to a specific situation or whether it is a personality trait that influences behaviour and thinking in general.

## Conclusion

Our results indicate that higher levels of GSE are associated with reduced odds of several health complaints, including anxiety, depression, and nervousness, and with favourable patterns of health-related behaviours. While GSE was significantly associated with only a limited number of chronic conditions, the observed relationships with health complaints and health-related behaviours suggest that GSE may serve as a broader psychological resource relevant to the onset, course, and day-to-day management of chronic conditions. Many of the health complaints examined also represent common symptoms of chronic diseases, which implies that higher GSE may help alleviate certain symptom manifestations and thereby contribute to improved psychological well-being. Improved psychological well-being, in turn, may facilitate more effective self-management of chronic conditions.

Most existing evidence linking self-efficacy to chronic conditions focuses on specific self-efficacy. However, our findings suggest that general self-efficacy may play a similar role at a broader, non-condition-specific level. Although GSE does not replace SSE in explaining disease-specific behaviours, the associations we identified indicate that GSE may constitute an overarching psychological factor that co-occurs with and potentially supports processes involved in symptom management and certain forms of adaptive health-related behaviours.

## Supplementary Information

Supplementary Material 1.

## Data Availability

Anonymized data, code, and other materials related to this study are available at the Open Science Framework (OSF) website under the following DOI: 10.17605/OSF.IO/5RM9X.

## Abbreviations

ADHD: Attention-deficit/Hyperactivity disorder
GSE: General Self-Efficacy
GSES: General Self-Efficacy Scale
OR: Odds Ratio
OUSHI: Olomouc University Social Health Institute
PC: Personal Computer
SE: Self-Efficacy
SSE: Specific Self-Efficacy
TV: Television
WHO: World Health Organization
95% CI: 95% Confidence Interval
