# Supplementary material for: “Is a general belief in one’s capabilities related to mental and physical health?”: The relationships between self-efficacy, health outcomes, and health-related behaviour
Source: BMC Psychol. 2026 Mar 5;14:510. doi: 10.1186/s40359-026-03996-7 (PMC13072497; doi:10.1186/s40359-026-03996-7)
Supplement: Supplementary file 1 — Supplementary Material 1. [file 40359_2026_3996_MOESM1_ESM.docx]

*Table 1:* *Monthly* *Frequency of Health-Related Behaviours*

| **Variable** | **Frequencies (%)** |
| --- | --- |
| Smoking | \| 1 \| : \| 2143 \| ( \| 67.8% \| ) \| \| --- \| --- \| --- \| --- \| --- \| --- \| \| 2 \| : \| 291 \| ( \| 9.2% \| ) \| \| 3 \| : \| 113 \| ( \| 3.6% \| ) \| \| 4 \| : \| 90 \| ( \| 2.8% \| ) \| \| 5 \| : \| 232 \| ( \| 7.3% \| ) \| \| 6 \| : \| 294 \| ( \| 9.3% \| ) \| |
| Drinking coffee | \| 1 \| : \| 548 \| ( \| 17.3% \| ) \| \| --- \| --- \| --- \| --- \| --- \| --- \| \| 2 \| : \| 370 \| ( \| 11.7% \| ) \| \| 3 \| : \| 289 \| ( \| 9.1% \| ) \| \| 4 \| : \| 378 \| ( \| 12.0% \| ) \| \| 5 \| : \| 1040 \| ( \| 32.9% \| ) \| \| 6 \| : \| 538 \| ( \| 17.0% \| ) \| |
| Drinking alcohol | \| 1 \| : \| 519 \| ( \| 16.4% \| ) \| \| --- \| --- \| --- \| --- \| --- \| --- \| \| 2 \| : \| 1385 \| ( \| 43.8% \| ) \| \| 3 \| : \| 713 \| ( \| 22.5% \| ) \| \| 4 \| : \| 447 \| ( \| 14.1% \| ) \| \| 5 \| : \| 86 \| ( \| 2.7% \| ) \| \| 6 \| : \| 13 \| ( \| 0.4% \| ) \| |
| Using TV or computer to relax | \| 1 \| : \| 143 \| ( \| 4.5% \| ) \| \| --- \| --- \| --- \| --- \| --- \| --- \| \| 2 \| : \| 213 \| ( \| 6.7% \| ) \| \| 3 \| : \| 410 \| ( \| 13.0% \| ) \| \| 4 \| : \| 629 \| ( \| 19.9% \| ) \| \| 5 \| : \| 1339 \| ( \| 42.3% \| ) \| \| 6 \| : \| 429 \| ( \| 13.6% \| ) \| |
| Using illegal drugs | \| 1 \| : \| 2855 \| ( \| 90.3% \| ) \| \| --- \| --- \| --- \| --- \| --- \| --- \| \| 2 \| : \| 204 \| ( \| 6.4% \| ) \| \| 3 \| : \| 43 \| ( \| 1.4% \| ) \| \| 4 \| : \| 30 \| ( \| 0.9% \| ) \| \| 5 \| : \| 21 \| ( \| 0.7% \| ) \| \| 6 \| : \| 10 \| ( \| 0.3% \| ) \| |

*Notes: N = 3,532; 1 = never, 2 = about once or twice, 3 = about every week, 4 = more than once a week, 5 = every day, 6 = many times a day*

*Table 2: Prevalence of Chronic Psychiatric* *Conditions*

| **Variable** | **Frequencies (%)** |
| --- | --- |
| ADHD | \| 1 \| : \| 3100 \| ( \| 96.9% \| ) \| \| --- \| --- \| --- \| --- \| --- \| --- \| \| 2 \| : \| 99 \| ( \| 3.1% \| ) \| |
| Anxiety | \| 1 \| : \| 2935 \| ( \| 91.7% \| ) \| \| --- \| --- \| --- \| --- \| --- \| --- \| \| 2 \| : \| 264 \| ( \| 8.3% \| ) \| |
| Paranoia | \| 1 \| : \| 3190 \| ( \| 99.7% \| ) \| \| --- \| --- \| --- \| --- \| --- \| --- \| \| 2 \| : \| 9 \| ( \| 0.3% \| ) \| |
| Depression | \| 1 \| : \| 3036 \| ( \| 94.9% \| ) \| \| --- \| --- \| --- \| --- \| --- \| --- \| \| 2 \| : \| 163 \| ( \| 5.1% \| ) \| |
| Schizotypal disorder | \| 1 \| : \| 3185 \| ( \| 99.6% \| ) \| \| --- \| --- \| --- \| --- \| --- \| --- \| \| 2 \| : \| 14 \| ( \| 0.4% \| ) \| |
| Paranoid schizophrenia | \| 1 \| : \| 3191 \| ( \| 99.7% \| ) \| \| --- \| --- \| --- \| --- \| --- \| --- \| \| 2 \| : \| 8 \| ( \| 0.3% \| ) \| |

*Notes: N = 3,532; 1 = Condition does not occur, 2 = conditions occurs*

*Table 3: Prevalence Chronic Conditions Table 4: Frequency of Health Complaints*

| **Variable** | **Frequencies (%)** |
| --- | --- |
| Asthma | \| 1 \| : \| 2891 \| ( \| 90.4% \| ) \| \| --- \| --- \| --- \| --- \| --- \| --- \| \| 2 \| : \| 308 \| ( \| 9.6% \| ) \| |
| Cancer | \| 1 \| : \| 3170 \| ( \| 99.1% \| ) \| \| --- \| --- \| --- \| --- \| --- \| --- \| \| 2 \| : \| 29 \| ( \| 0.9% \| ) \| |
| Stroke | \| 1 \| : \| 3192 \| ( \| 99.8% \| ) \| \| --- \| --- \| --- \| --- \| --- \| --- \| \| 2 \| : \| 7 \| ( \| 0.2% \| ) \| |
| Obesity | \| 1 \| : \| 2940 \| ( \| 91.9% \| ) \| \| --- \| --- \| --- \| --- \| --- \| --- \| \| 2 \| : \| 259 \| ( \| 8.1% \| ) \| |
| Migraine | \| 1 \| : \| 2946 \| ( \| 92.1% \| ) \| \| --- \| --- \| --- \| --- \| --- \| --- \| \| 2 \| : \| 253 \| ( \| 7.9% \| ) \| |
| Diabetes | \| 1 \| : \| 3123 \| ( \| 97.6% \| ) \| \| --- \| --- \| --- \| --- \| --- \| --- \| \| 2 \| : \| 76 \| ( \| 2.4% \| ) \| |
| Allergies | \| 1 \| : \| 773 \| ( \| 24.2% \| ) \| \| --- \| --- \| --- \| --- \| --- \| --- \| \| 2 \| : \| 2426 \| ( \| 75.8% \| ) \| |
| Arthritis | \| 1 \| : \| 3113 \| ( \| 97.3% \| ) \| \| --- \| --- \| --- \| --- \| --- \| --- \| \| 2 \| : \| 86 \| ( \| 2.7% \| ) \| |
| Backpain | \| 1 \| : \| 2665 \| ( \| 83.3% \| ) \| \| --- \| --- \| --- \| --- \| --- \| --- \| \| 2 \| : \| 534 \| ( \| 16.7% \| ) \| |
| Psoriasis | \| 1 \| : \| 3157 \| ( \| 98.7% \| ) \| \| --- \| --- \| --- \| --- \| --- \| --- \| \| 2 \| : \| 42 \| ( \| 1.3% \| ) \| |
| Pelvic pain | \| 1 \| : \| 3030 \| ( \| 94.7% \| ) \| \| --- \| --- \| --- \| --- \| --- \| --- \| \| 2 \| : \| 169 \| ( \| 5.3% \| ) \| |
| Hypertension | \| 1 \| : \| 2968 \| ( \| 92.8% \| ) \| \| --- \| --- \| --- \| --- \| --- \| --- \| \| 2 \| : \| 231 \| ( \| 7.2% \| ) \| |
| Skin diseases | \| 1 \| : \| 2913 \| ( \| 91.1% \| ) \| \| --- \| --- \| --- \| --- \| --- \| --- \| \| 2 \| : \| 286 \| ( \| 8.9% \| ) \| |
| Gastric reflux | \| 1 \| : \| 3048 \| ( \| 95.3% \| ) \| \| --- \| --- \| --- \| --- \| --- \| --- \| \| 2 \| : \| 151 \| ( \| 4.7% \| ) \| |
| Thyroid disease | \| 1 \| : \| 2980 \| ( \| 93.2% \| ) \| \| --- \| --- \| --- \| --- \| --- \| --- \| \| 2 \| : \| 219 \| ( \| 6.8% \| ) \| |
| Chronic tiredness | \| 1 \| : \| 3125 \| ( \| 97.7% \| ) \| \| --- \| --- \| --- \| --- \| --- \| --- \| \| 2 \| : \| 74 \| ( \| 2.3% \| ) \| |
| Pain of unclear origin | \| 1 \| : \| 3100 \| ( \| 96.9% \| ) \| \| --- \| --- \| --- \| --- \| --- \| --- \| \| 2 \| : \| 99 \| ( \| 3.1% \| ) \| |
| Chronic lung disease | \| 1 \| : \| 3173 \| ( \| 99.2% \| ) \| \| --- \| --- \| --- \| --- \| --- \| --- \| \| 2 \| : \| 26 \| ( \| 0.8% \| ) \| |

| **Variable** | **Frequencies (%)** |
| --- | --- |
| Trouble falling asleep | \| 1 \| : \| 968 \| ( \| 29.9% \| ) \| \| --- \| --- \| --- \| --- \| --- \| --- \| \| 2 \| : \| 999 \| ( \| 30.8% \| ) \| \| 3 \| : \| 522 \| ( \| 16.1% \| ) \| \| 4 \| : \| 464 \| ( \| 14.3% \| ) \| \| 5 \| : \| 286 \| ( \| 8.8% \| ) \| |
| Nervousness | \| 1 \| : \| 573 \| ( \| 17.7% \| ) \| \| --- \| --- \| --- \| --- \| --- \| --- \| \| 2 \| : \| 1231 \| ( \| 38.0% \| ) \| \| 3 \| : \| 698 \| ( \| 21.5% \| ) \| \| 4 \| : \| 518 \| ( \| 16.0% \| ) \| \| 5 \| : \| 219 \| ( \| 6.8% \| ) \| |
| Dizziness | \| 1 \| : \| 2467 \| ( \| 76.2% \| ) \| \| --- \| --- \| --- \| --- \| --- \| --- \| \| 2 \| : \| 485 \| ( \| 15.0% \| ) \| \| 3 \| : \| 154 \| ( \| 4.8% \| ) \| \| 4 \| : \| 92 \| ( \| 2.8% \| ) \| \| 5 \| : \| 41 \| ( \| 1.3% \| ) \| |
| Headache | \| 1 \| : \| 714 \| ( \| 22.0% \| ) \| \| --- \| --- \| --- \| --- \| --- \| --- \| \| 2 \| : \| 1681 \| ( \| 51.9% \| ) \| \| 3 \| : \| 493 \| ( \| 15.2% \| ) \| \| 4 \| : \| 283 \| ( \| 8.7% \| ) \| \| 5 \| : \| 68 \| ( \| 2.1% \| ) \| |
| Stomachache | \| 1 \| : \| 1580 \| ( \| 48.8% \| ) \| \| --- \| --- \| --- \| --- \| --- \| --- \| \| 2 \| : \| 1114 \| ( \| 34.4% \| ) \| \| 3 \| : \| 293 \| ( \| 9.0% \| ) \| \| 4 \| : \| 197 \| ( \| 6.1% \| ) \| \| 5 \| : \| 55 \| ( \| 1.7% \| ) \| |
| Intestinal complaints | \| 1 \| : \| 1593 \| ( \| 49.2% \| ) \| \| --- \| --- \| --- \| --- \| --- \| --- \| \| 2 \| : \| 1120 \| ( \| 34.6% \| ) \| \| 3 \| : \| 283 \| ( \| 8.7% \| ) \| \| 4 \| : \| 184 \| ( \| 5.7% \| ) \| \| 5 \| : \| 59 \| ( \| 1.8% \| ) \| |
| Irritability, bad mood | \| 1 \| : \| 468 \| ( \| 14.4% \| ) \| \| --- \| --- \| --- \| --- \| --- \| --- \| \| 2 \| : \| 1373 \| ( \| 42.4% \| ) \| \| 3 \| : \| 752 \| ( \| 23.2% \| ) \| \| 4 \| : \| 511 \| ( \| 15.8% \| ) \| \| 5 \| : \| 135 \| ( \| 4.2% \| ) \| |
| Heart pounding, chest pain | \| 1 \| : \| 2013 \| ( \| 62.1% \| ) \| \| --- \| --- \| --- \| --- \| --- \| --- \| \| 2 \| : \| 718 \| ( \| 22.2% \| ) \| \| 3 \| : \| 290 \| ( \| 9.0% \| ) \| \| 4 \| : \| 166 \| ( \| 5.1% \| ) \| \| 5 \| : \| 52 \| ( \| 1.6% \| ) \| |
| Tingling in limbs or face | \| 1 \| : \| 2299 \| ( \| 71.0% \| ) \| \| --- \| --- \| --- \| --- \| --- \| --- \| \| 2 \| : \| 573 \| ( \| 17.7% \| ) \| \| 3 \| : \| 189 \| ( \| 5.8% \| ) \| \| 4 \| : \| 123 \| ( \| 3.8% \| ) \| \| 5 \| : \| 55 \| ( \| 1.7% \| ) \| |

| Ischemic heart disease | \| 1 \| : \| 3166 \| ( \| 99.0% \| ) \| \| --- \| --- \| --- \| --- \| --- \| --- \| \| 2 \| : \| 33 \| ( \| 1.0% \| ) \| |
| --- | --- | --- | --- | --- | --- | --- | --- | --- | --- | --- | --- | --- | --- |
| Inflammatory bowel disease | \| 1 \| : \| 3154 \| ( \| 98.6% \| ) \| \| --- \| --- \| --- \| --- \| --- \| --- \| \| 2 \| : \| 45 \| ( \| 1.4% \| ) \| |
| Stomach or duodenal ulcers | \| 1 \| : \| 3171 \| ( \| 99.1% \| ) \| \| --- \| --- \| --- \| --- \| --- \| --- \| \| 2 \| : \| 28 \| ( \| 0.9% \| ) \| |

| Feelings of depression | \| 1 \| : \| 899 \| ( \| 27.8% \| ) \| \| --- \| --- \| --- \| --- \| --- \| --- \| \| 2 \| : \| 1086 \| ( \| 33.5% \| ) \| \| 3 \| : \| 620 \| ( \| 19.1% \| ) \| \| 4 \| : \| 453 \| ( \| 14.0% \| ) \| \| 5 \| : \| 181 \| ( \| 5.6% \| ) \| |
| --- | --- | --- | --- | --- | --- | --- | --- | --- | --- | --- | --- | --- | --- | --- | --- | --- | --- | --- | --- | --- | --- | --- | --- | --- | --- | --- | --- | --- | --- | --- | --- |
| Sore throat, cold | \| 1 \| : \| 1496 \| ( \| 46.2% \| ) \| \| --- \| --- \| --- \| --- \| --- \| --- \| \| 2 \| : \| 1336 \| ( \| 41.2% \| ) \| \| 3 \| : \| 220 \| ( \| 6.8% \| ) \| \| 4 \| : \| 142 \| ( \| 4.4% \| ) \| \| 5 \| : \| 45 \| ( \| 1.4% \| ) \| |

*Notes: N = 3,532; 1 = Condition does not occur, 2 = conditions occurs*

*Notes: N = 3,532; 1 = never, 2 = about once or twice, 3 = about every week, 4 = more than once a week, 5 = every day*
